# Key sequence features of CRISPR RNA for dual-guide CRISPR-Cas9 ribonucleoprotein complexes assembled with wild-type or HiFi Cas9

**DOI:** 10.1093/nar/gkac100

**Published:** 2022-02-15

**Authors:** Keita Okada, Kanae Aoki, Teruyuki Tabei, Kota Sugio, Katsunori Imai, Yuki Bonkohara, Yusuke Kamachi

**Affiliations:** School of Environmental Science and Engineering, Kochi University of Technology, Kami, Kochi 782-8502, Japan; School of Environmental Science and Engineering, Kochi University of Technology, Kami, Kochi 782-8502, Japan; School of Environmental Science and Engineering, Kochi University of Technology, Kami, Kochi 782-8502, Japan; School of Environmental Science and Engineering, Kochi University of Technology, Kami, Kochi 782-8502, Japan; School of Environmental Science and Engineering, Kochi University of Technology, Kami, Kochi 782-8502, Japan; School of Environmental Science and Engineering, Kochi University of Technology, Kami, Kochi 782-8502, Japan; School of Environmental Science and Engineering, Kochi University of Technology, Kami, Kochi 782-8502, Japan

## Abstract

Specific sequence features of the protospacer and protospacer-adjacent motif (PAM) are critical for efficient cleavage by CRISPR-Cas9, but current knowledge is largely derived from single-guide RNA (sgRNA) systems assessed in cultured cells. In this study, we sought to determine gRNA sequence features of a more native CRISPR-Cas9 ribonucleoprotein (RNP) complex with dual-guide RNAs (dgRNAs) composed of crRNA and tracrRNA, which has been used increasingly in recent CRISPR-Cas9 applications, particularly in zebrafish. Using both wild-type and HiFi SpCas9, we determined on-target cleavage efficiencies of 51 crRNAs in zebrafish embryos by assessing indel occurrence. Statistical analysis of these data identified novel position-specific mononucleotide features relevant to cleavage efficiencies throughout the protospacer sequence that may be unique to CRISPR-Cas9 RNPs pre-assembled with perfectly matched gRNAs. Overall features for wild-type Cas9 resembled those for HiFi Cas9, but specific differences were also observed. Mutational analysis of mononucleotide features confirmed their relevance to cleavage efficiencies. Moreover, the mononucleotide feature-based score, CRISPR-kp, correlated well with efficiencies of gRNAs reported in previous zebrafish RNP injection experiments, as well as independently tested crRNAs only in RNP format, but not with Cas9 mRNA co-injection. These findings will facilitate design of gRNA/crRNAs in genome editing applications, especially when using pre-assembled RNPs.

## INTRODUCTION

Clustered regularly interspaced short palindromic repeat (CRISPR) and CRISPR-associated protein (Cas) systems function as adaptive immune systems of bacteria and archaea against foreign nucleic acids by acting as sequence-specific nucleases (1,2). The CRISPR-Cas9 nuclease system has been utilized in various genome editing studies due to its highly programmable nature and ease of use (3–6). In its natural form, the CRISPR-Cas9 system is composed of Cas9 protein and guide RNA (gRNA) consisting of two RNA molecules, CRISPR RNA (crRNA) and trans-activating crRNA (tracrRNA) (1,2). With this system, theoretically, any genomic sequence can be edited by simply changing the crRNA molecule that recognizes a 20-nucleotide (nt) target protospacer sequence associated with the PAM [protospacer adjacent motif; NGG for *Streptococcus pyogenes* Cas9 (SpCas9)].

To simplify the CRISPR-Cas9 system, Jinek *et al.* (4) fused crRNA and tracrRNA to create a single RNA chimera called single-guide RNA (sgRNA), since it is easily expressed from a single promoter for either *in vivo* or *in vitro* transcription. Since then, the sgRNA system has been predominantly used for genome editing in cultured cells and various model organisms, including zebrafish (3,7). In recent years, however, a more natural dual-guide RNA (dgRNA) system using chemically synthesized RNAs has been increasingly employed because it offers greater RNA stability, resulting from chemical modifications. Additionally, it has editing activity similar to or even higher than the sgRNA system, and most importantly, it is not encumbered by restrictions on target sequence selection associated with transcriptional initiation by RNA polymerases (8–10).

For *in vitro* synthesis of sgRNAs, T7 RNA polymerase-mediated transcription is generally used, in which extra guanine nucleotides are often added at the 5′ end as a requirement of the T7 RNA polymerase. However, a recent zebrafish study indicated that these supernumerary guanine nucleotides are deleterious to CRISPR-Cas9 activity (9), although 5′-end mismatches are thought to be tolerated for cleavage (11), suggesting an advantage of the dgRNA system for zebrafish genome editing. Similarly, the U6 promoter that is used for *in vivo* sgRNA synthesis requires guanine as the first nucleotide for transcription by RNA Polymerase III (Pol III), which also reduces flexibility in target sequence selection (5,6). Alternatively, when the nucleotide at the 5′ end of the protospacer is A, T or C, an sgRNA starting with a mismatched guanine is used (generally called a gN19-NGG target), which may also affect gRNA activity (12). In recent years, high-fidelity Cas9 variants have been developed to avoid off-target effects. For such variants, it has been shown that sgRNAs harboring perfectly matched 20-nt guide sequences are required for efficient cleavage (13), suggesting that the dgRNA system is particularly advantageous when used with high-fidelity Cas9 variants.

The success of genome editing depends on an efficient gRNA with high nuclease activity, as genomic DNA cleavage is a prerequisite for repair-mediated gene knockout and knock-in in most CRISPR-Cas9 applications. Therefore, it is critical to carefully select a gRNA with high on-target cleavage activity. To predict cleavage efficiency, gRNA sequence features that could affect CRISPR-Cas9 cleavage activity have been explored and various gRNA design tools have been developed based on these analyses (12,14–26). In these and other studies, several strategies have been used to evaluate efficiency of gRNAs, which are categorized as indirect or direct when measuring frequencies of CRISPR-Cas9-induced insertions and deletions (indels). The former approach includes selection of active gRNAs through phenotypic alteration, such as loss of surface marker expression (14) and indirect estimation based upon reduction of luciferase reporter gene expression (23) or measurement of reconstituted activities of luciferase or fluorescent genes (27), whereas the latter approach directly analyzes indel frequencies using high-throughput sequencing (16,17) or Sanger sequencing-based methods, called Tracking of Indels by DEcomposition (TIDE) (28) and Inference of CRISPR Edits (ICE) (29). Among sequencing-based methods, high-throughput sequencing of a PCR amplicon derived from edited genomic DNA surrounding the CRISPR-Cas9 target site is the most straightforward method for measuring indel frequencies, although it is labor-intensive and costly. To overcome this problem, Sanger sequencing-based TIDE and ICE were developed, in which indels are assessed computationally by comparing Sanger sequence traces of PCR amplicons derived from edited and unedited genomic DNAs. Indel frequencies obtained from TIDE and ICE exhibit significant correlations with those derived from high-throughput sequencing (28–30). Although indirect methods appear to be useful to find crRNA features related to gene knockout, it is unclear how precisely they measure indel frequencies themselves, suggesting that direct methods are advantageous for precise evaluation of gRNA cleavage efficiency (31).

The aforementioned studies have identified gRNA sequence features that affect CRISPR-Cas9 cleavage activity. These features include composition of nucleotides at specific positions (generally, position 1 is assigned to the 5′-nucleotide of the 20-nt protospacer sequence, and positions 21–23 correspond to PAM) (12,14,16–18,22,24,25), GC content (15,24,32,33), secondary structures (34–36), and chromatin accessibility (34,37). Based on these features, various gRNA design tools have been developed to predict on-target cleavage activity, but these are all based on experiments performed using sgRNAs on specific cell types or model organisms; thus, they may only be applicable to similar experimental conditions, particularly the choice of sgRNA expression systems, such as the U6 or T7 promoter (20). The U6 promoter has been commonly used in cultured cell-based CRISPR-Cas9 systems, where 5′-end mismatched gRNAs are occasionally used and sgRNA expression levels vary due to RNA stability and/or premature termination of Pol III-driven transcription (15,38). Similarly, *in vivo* RNA stability of sgRNA generated from the T7 promoter was reported as one of the major determinants of gRNA efficiency in zebrafish (17). In contrast, dgRNA is generally used as a pre-assembled CRISPR-Cas9 RNP complex, in which the effect of RNA stability is expected to be minimal. For these reasons, currently recognized sequence features may be biased toward characteristics of sgRNA expression systems (20).

One major concern about the CRISPR-Cas9 system is off-target cleavage. To overcome this problem, engineered Cas9 variants with higher specificity have been developed (39–45). Recent studies on these high-fidelity Cas9 variants have revealed that they have slightly different nucleotide preferences. Since high-fidelity Cas9 variants require perfectly matched 20-nt protospacer sequences for high on-target activity (13), gRNA sequence features for high-fidelity Cas9 variants should ideally be analyzed using perfectly matched gRNAs.

Therefore, this study sought to answer the following questions: Are sequence features identified for sgRNA similar to those for crRNA in the dgRNA CRISPR-Cas9 RNP system? How similar are sequence features of wild-type (WT) and high-fidelity variant HiFi Cas9 (39) in the dgRNA CRISPR-Cas9 RNP system? How precisely do current design tools predict on-target activity of crRNA in the dgRNA RNP system, particularly in zebrafish? To this end, using WT and HiFi Cas9, we determined on-target cleavage efficiencies of 51 crRNAs in the form of the crRNA-tracrRNA-Cas9 ribonucleoprotein complexes in zebrafish embryos, using TIDE and ICE tools. Statistical analysis of nucleotide compositions of crRNAs revealed that specific mononucleotides at particular positions significantly affected cleavage activity. Some of these have not been identified in previous studies, suggesting the existence of perfectly matched gRNA/dgRNA and/or pre-assembled RNP specific features. Moreover, these sequence features differ slightly between WT and HiFi Cas9. Furthermore, we experimentally confirmed the importance of mononucleotide features on crRNA activity through mutational analysis. Importantly, our statistical *P*-value-based scoring was found to correlate well with cleavage efficiencies of our independent crRNA set and those of gRNAs in RNP reported in previous studies. When we compared cleavage efficiency values with scores predicted by available gRNA design tools, some gRNA design tools, particularly those developed using deep learning, were useful for crRNAs. These results suggest that gRNA sequence features that determine cleavage efficiency may differ slightly among variants of the CRISPR-Cas9 system and that incorporating these parameters into gRNA design will further improve the utility of CRISPR-Cas9 in genome editing.

## MATERIALS AND METHODS

### Zebrafish husbandry

Zebrafish (*Danio rerio*) were bred and maintained under standard laboratory conditions on a 14 h/10 h light/dark cycle. All zebrafish experiments were conducted in accordance with the Fundamental Guidelines for Proper Conduct of Animal Experiments and Related Activities in Academic Research Institutions under the jurisdiction of the Ministry of Education, Culture, Sports, Science and Technology of Japan, using protocols approved by the Animal Experiments Committee of Kochi University of Technology.

### Preparation of CRISPR-Cas9 RNP complexes

CRISPR-Cas9 RNP complexes were prepared using crRNAs (Alt-R CRISPR-Cas9 crRNA) (listed in Supplementary Table S1), tracrRNA (Alt-R CRISPR-Cas9 tracrRNA-ATTO 550), and Cas9 protein (Alt-R S.p. Cas9 Nuclease V3 and HiFi Cas9 Nuclease V3) purchased from IDT, and used according to the manufacturer's protocol (Zebrafish embryo microinjection: Ribonucleoprotein delivery using the Alt-R CRISPR-Cas9 System). In brief, 100 μM crRNA and 100 μM tracrRNA were mixed in Nuclease-Free Duplex Buffer (IDT) to create a 3 μM gRNA solution. The solution was heated at 95°C for 5 min and then cooled to room temperature. The 3 μM gRNA solution was combined with an equimolar amount of 3 μM Cas9 diluted in Cas9 working buffer (20 mM HEPES pH 7.5, 150 mM KCl) and incubated at 37°C for 10 min to assemble 1.5 μM RNP complex.

### Preparation of HiFi Cas9 mRNA

pCS2-HiFi Cas9 was constructed by inserting a HiFi Cas9-encoding DNA fragment from pX330-Flag-HiFi SpCas9 (a gift from Ervin Welker, Addgene plasmid #126778) into the pCS2 vector. pCS2-HiFi Cas9 was linearized with NotI and used as a template for mRNA synthesis using AmpliCap SP6 High Yield Message Maker Kit (CELLSCRIPT). mRNA was then purified with RNA clean & concentrator (Zymo Research). HiFi Cas9 mRNA solution (200 ng/μl) was combined with the same volume of the 3 μM gRNA solution and used for microinjection.

### Microinjection and genomic DNA preparation

Typically, 1 nl of 1.5 μM CRISPR-Cas9 RNP complex was microinjected into the yolk of 1-cell stage embryos of TL zebrafish, delivering 1.5 fmol of RNP complex. When HiFi Cas9 mRNA was used, 2 nl of solution containing 100 ng/μl HiFi Cas9 mRNA and 1.5 μM gRNA were typically microinjected, delivering 200 pg of Cas9 mRNA and 3 fmol of gRNA. Injected embryos were raised at 28°C until 24 h post-fertilization (hpf) and genomic DNA was prepared from pools of embryos using 20 μl per embryo of DNA extraction buffer (low EDTA) (10 mM Tris–HCl pH 8, 0.1 mM EDTA, 0.2% Triton X-100, 200 μg/ml Proteinase K). After incubation at 55°C for 2–3 h with occasional vortexing and heating at 95°C for 10 min to inactivate Protease K, crude genomic DNA solution was used directly in polymerase chain reaction (PCR) assays.

### Assessment of indel frequencies using TIDE and ICE tools

Quantification of indel frequencies was performed using the tracking-of-indels-by-decomposition (TIDE) algorithm (https://tide.nki.nl) and inference-of-CRISPR-edits (ICE) method (https://ice.synthego.com), both of which were developed to analyze indels using Sanger sequence traces generated from PCR amplicons of target DNA regions (28,29). PCR primers were designed to amplify a fragment of approximately 600 bp surrounding the CRISPR-Cas9 target site (Supplementary Table S2). PCR was performed using Taq DNA Polymerase (New England Biolabs, NEB) in 30 μl of reaction mixture containing 1× ThermoPol Reaction Buffer, 200 μM dNTPs, 0.5 μM forward and reverse primers, 0.15 embryo equivalent of genomic DNA, 0.75 units of Taq DNA Polymerase, and 1× red sucrose solution (10% sucrose, 0.17 mM cresol red). PCR conditions were as follows: initial denaturation at 95°C for 30 s; 30 cycles of denaturation at 95°C for 15 s, annealing at 60°C or 65°C for 30 s, and extension at 68°C for 45 s; final extension at 68°C for 5 min. After confirming amplification of PCR products by agarose gel electrophoresis, PCR products were purified using NucleoSpin Gel and PCR Clean-up (Macherey-Nagel) or KAPA Pure Beads (Kapa Biosystems). Purified PCR products were Sanger sequenced using primers listed in Supplementary Table S2. Sanger sequencing traces from genomic DNAs prepared from CRISPR-Cas9 injected and uninjected control embryos were then used for TIDE (version 3.2.0; alignment window (bp) = 25, Indel size range = 25) and ICE (v2) to assess indel frequencies.

### *In vitro* CRISPR-Cas9 cleavage assay

Purified PCR products that were amplified from TL zebrafish genomic DNA with primers for the TIDE and ICE assays were used as cleavage assay substrates. The *in vitro* cleavage reaction was performed according to the IDT protocol (Alt-R CRISPR-Cas9 System: *In vitro* cleavage of target DNA with ribonucleoprotein complex) in 10 μl of reaction mixture containing Cas9 Nuclease Reaction Buffer (20 mM HEPES pH 6.5, 100 mM NaCl, 5 mM MgCl_2_, 0.1 mM EDTA), 100 nM CRISPR-Cas9 RNP complex, and 5 nM PCR products. The reaction mixture was incubated at 37°C for 20 min. The cleavage reaction was terminated by adding 1 μl of 20 mg/ml Proteinase K and incubating at 56°C for 10 min. The DNA substrate was subjected to agarose gel electrophoresis, and the gel was stained with 1× dsGreen (Lumiprobe) and visualized with a Fusion imaging system (Vilber Lourmat). Band intensities were quantified using ImageJ (v1.53) and cleavage efficiency was calculated by quantifying band intensities of uncleaved fragments.

### Sequence feature analysis by kpLogo

Sequence features of crRNAs were analyzed with the kpLogo (k-mer probability logo) tool using the locally installed program with default option settings (46) (http://kplogo.wi.mit.edu/, https://github.com/xuebingwu/kpLogo v1.1). Protospacer and PAM sequences of the tested 51 crRNAs that were weighted by cleavage frequencies obtained with ICE were used as input data.

### *In vivo* plasmid cleavage assay

Genomic DNA fragments of the TL zebrafish strain that encompass target sites of crRNAs (otx2b_AA/AB, pax2a_AJ and sox19a-KO_4) were amplified using KOD-Plus-Neo DNA polymerase (Toyobo) with the primers listed in Supplementary Table S2. These fragments were cloned into the pUC19 vector at EcoRI and either HindIII or PstI restriction sites and used as CRISPR-Cas9 substrates. Mutant target plasmids for mutant crRNAs were constructed by site-directed mutagenesis using overlap extension PCR, in which PCRs were performed using KOD-One PCR Master Mix (Toyobo) with specific primers containing the intended mutations (Supplementary Table S2) and the wild-type target plasmids as templates.

1 nl of the 10 ng/μl plasmid DNA (10 pg) was first microinjected into the cytoplasm of 1-cell stage embryos and 2 nl of 1.5 μM RNP complex (3 fmol) were subsequently microinjected into the yolk of plasmid-injected embryos. Injected embryos were raised at 28°C for 24 h and DNAs were prepared from both the nuclear and cytoplasmic fractions (47). In brief, 20 embryos were dechorionated using tweezers in E2 embryo medium (48) and washed 3x in 1 ml of ice-cold egg lysis buffer (125 mM NaCl, 5 mM MgCl_2_, 100 mM glycine, 20 mM HEPES pH 7.6) on ice. Embryos were homogenized with a micro pestle in 20 μl of egg lysis buffer on ice. Homogenate was centrifuged at 10 000 × g for 8 min at 4°C, and the supernatant was used as a cytoplasmic fraction. The pellet that was resuspended in 200 μl of ice-cold egg lysis buffer was underlaid with 200 μl of ice-cold 1 M sucrose/PBS and centrifuged at 5000 × g for 8 min at 4°C. This process was repeated once and the pellet was used as a nuclear fraction. DNA extraction buffer (low EDTA) was added to both fractions to achieve 0.05 embryo equivalent/μl and lysates were incubated at 55°C for 2–3 h with occasional mixing and heated at 95°C for 10 min to inactivate Proteinase K. Indel frequencies were assessed using TIDE, as described above, in which a fragment of approximately 600 bp surrounding the CRISPR-Cas9 target site on the plasmid was amplified using primers specific for the plasmid backbone sequence (Supplementary Table S2).

### CRISPR-Cas9 gRNA design tools

CRISPR-Cas9 gRNA design tools used in this study are listed in Supplementary Table S3, which summarizes their availability, experimental conditions to generate data (organism/cell types and gRNA types), and modelling methods.

## RESULTS

### Evaluation of cleavage efficiency of CRISPR-Cas9 crRNAs in zebrafish embryos

Cleavage efficiency is one of the most critical prerequisites for CRISPR-Cas9 experiments. Importantly, a recent zebrafish study showed that the CRISPR-Cas9 ribonucleoprotein (RNP) complex composed of chemically synthesized crRNA, tracrRNA and Cas9 protein is more efficient than one composed of a single-guide RNA (sgRNA, a fusion of crRNA and tracrRNA) and Cas9 protein, due to adverse effects of supernumerary guanine (G) nucleotides at 5′ ends that are required for T7 RNA polymerase-mediated sgRNA synthesis (9). The U6 promoter-based sgRNA expression system, which has been predominantly used in genome editing in cultured cells, also requires a G nucleotide at the 5′ end for transcription, which potentially reduces cleavage activity of CRISPR-Cas9 (12,22). Although several gRNA design tools have been developed based on factors that potentially affect cleavage efficiency and have been used for various CRISPR-Cas9 experiments, published tools are all based on experiments using sgRNA systems; thus, it is currently unclear whether they can precisely predict cleavage efficiency of crRNAs in the synthetic dual-guide RNA (dgRNA) system. Therefore, in this study, we systematically evaluated cleavage efficiencies of crRNAs that were pre-assembled *in vitro* into RNP complexes in order to extract sequence features that might be specific to the dgRNA CRISPR-Cas9 system, perfectly matched gRNAs, and/or the RNP form. To this end, we designed 51 crRNAs targeting 17 gene loci, including three crRNAs used in our previous study (49), in which target sites were located around the start or stop codons for future knock-in experiments or within coding sequences for future knockout experiments. crRNA selection performed using the IDT design tool prioritized higher off-target scores (i.e. lower off-target risk), whereas the selected crRNAs exhibited a wide range of on-target scores (Supplementary Table S1).

**Figure 1.**
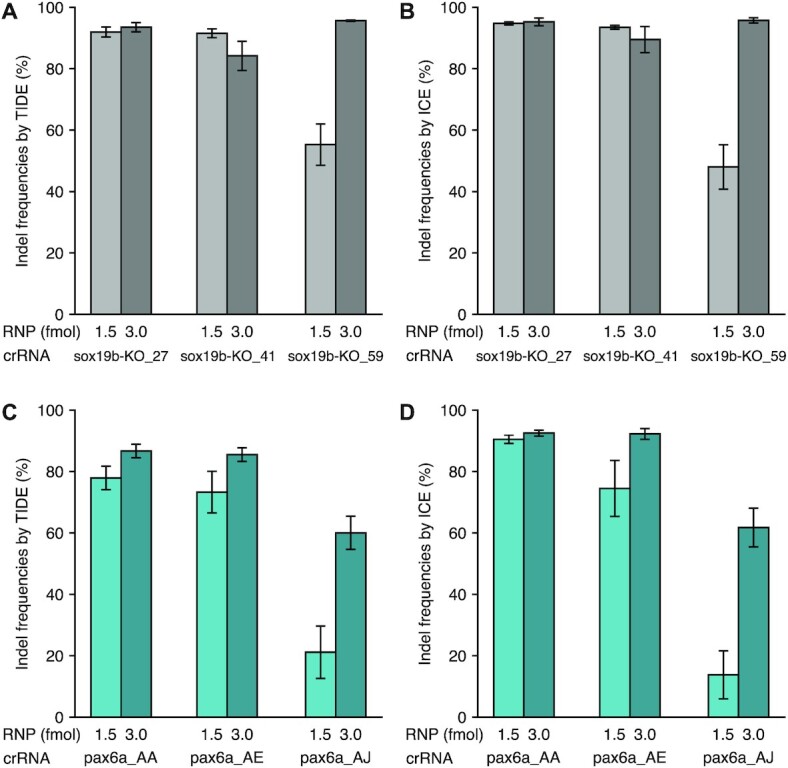
Establishing adequate quantities of CRISPR-Cas9 RNP complexes for crRNA evaluation. (**A**, **B**) Indel frequencies (in %) generated by different amounts of RNP complex with WT Cas9. 1 nl and 2 nl of 1.5 μM RNP complex (1.5 and 3 fmol, respectively) assembled using WT Cas9 with the indicated crRNAs were microinjected into 1-cell stage embryos, and indel frequencies were assessed with TIDE (A) and ICE (B). (**C**, **D**) Indel frequencies (in %) generated by different amounts of RNP complex with HiFi Cas9. TIDE (C) and ICE (D) were used for the assessment.

We sought to determine the cleavage efficiency of crRNAs assembled with both WT and HiFi Cas9, since HiFi Cas9 has not been well characterized, despite increasing demand for it in medical applications (50). We used the Sanger sequence-based methods, TIDE and ICE, to determine insertion-deletion (indel) frequencies. These methods estimate frequencies of indel occurrence by decomposition of Sanger trace data and output indel frequency values, comparable to those obtained with high-throughput sequencing (28,29). We first determined an adequate amount of the CRISPR-Cas9 RNP complex, with which crRNAs were expected to show gradual low-to-high cleavage efficiencies. For this, 1.5 or 3 fmol of RNP complex assembled using WT or HiFi Cas9 were microinjected into 1-cell stage embryos using three crRNAs, and genomic DNA was prepared from four pools of five embryos at 24 hpf. The target region was PCR-amplified from uninjected controls and injected embryo DNA and its Sanger-sequenced trace data were used to assess cleavage efficiencies with TIDE and ICE. The 1.5-fmol injections resulted in different indel frequencies among the three crRNAs, whereas the 3-fmol injections all exhibited higher indel frequencies with small differences, suggesting that the 1.5-fmol RNP injection is more useful to distinguish low-to-high levels of crRNA efficiencies (Figure 1). Notably, this amount was approximately one third of that used by Hoshijima *et al.* to achieve maximal mutagenesis in zebrafish embryos (9).

**Figure 2.**
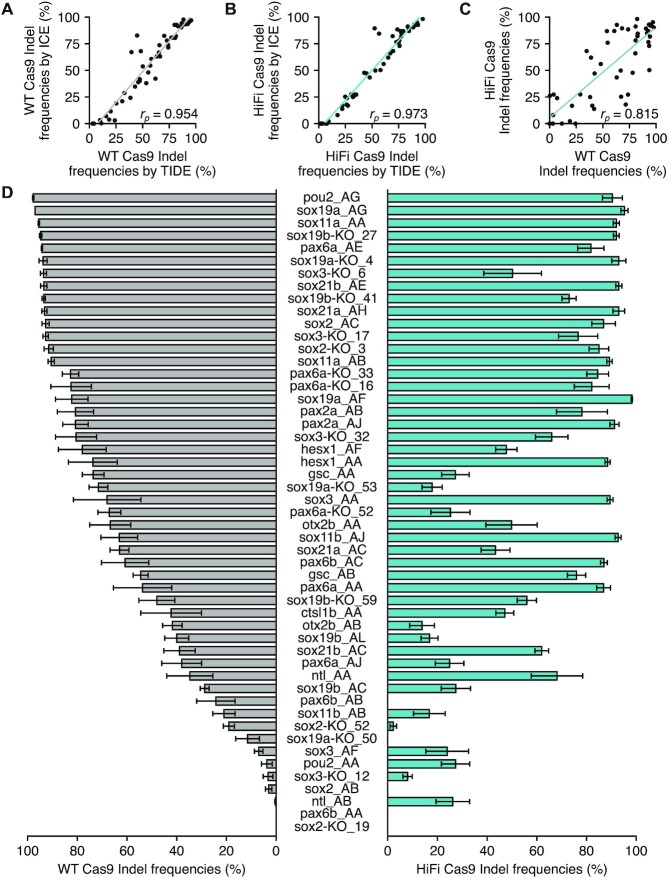
Cleavage efficiencies of the 51 crRNAs in the dgRNA RNP complex assembled with WT or HiFi Cas9. (**A**) A scatter plot of indel frequencies (in %) of the 51 crRNAs assessed with TIDE and ICE with the use of WT Cas9. (**B**) A scatter plot of indel frequencies of the 51 crRNAs assessed with TIDE and ICE using HiFi Cas9. (**C**) Comparison of indel frequencies generated with WT and HiFi Cas9 RNP complexes and assessed with ICE. Pearson correlation coefficients (*r*_p_) are shown in each panel (A–C). (**D**) Indel frequencies (in %) assessed with ICE for the 51 crRNAs in dgRNA RNP complexes assembled with WT Cas9 and HiFi Cas9 are plotted on the left and right sides, respectively. Means of replicates are shown with the standard error bars. The experimental dataset for (A)–(D) is shown in Supplementary Table S4.

Using these experimental conditions, 51 crRNAs were assessed for their cleavage efficiencies using both WT and HiFi Cas9 (Figure 2 and Supplementary Table S4). We generally used four pools of five injected embryos for TIDE and ICE analyses, but six or more pooled embryo samples were analyzed when a particular crRNA exhibited more variable indel frequencies. Indel frequencies calculated using TIDE and ICE were well correlated (Figure 2A and B); however, TIDE appeared to be able to detect low frequencies of indels that ICE missed, while TIDE outputted much lower values than ICE for a few samples, probably when parameters may not have been optimal. Thus, in the following experiments, we generally used ICE to evaluate crRNAs, whereas TIDE was used when low levels of indel frequencies were to be assessed. Indel frequencies assessed with ICE ranged from 0 to near 100% for both WT and HiFi Cas9 (Figure 2C and D), in which average values were 58.9% and 55.8%, respectively, consistent with previous studies showing comparable cleavage activity between WT and HiFi Cas9 in either form of RNP complex assembled *in vitro* with dgRNA (39) or assembled *in vivo* with sgRNA (45). Among the 51 crRNAs, 21 and 23 crRNAs exhibited > 75% indel frequencies for WT and HiFi Cas9, respectively, even with the use of less RNP complex in our assay, indicating that the dgRNA CRISPR-Cas9 system generally works efficiently in zebrafish embryos. However, some crRNAs (11 for WT and 13 for HiFi Cas9) showed low cleavage efficiency with < 25% indel frequencies. The Pearson correlation coefficient between the WT and HiFi Cas9 values was 0.815 (Figure 2C), suggesting that they have similar sequence preferences for crRNA. However, some crRNAs such as sox19a-KO_53 and gsc_AA exhibited divergent cleavage activities when compared between WT and HiFi Cas9 PNPs, suggesting different sequence preferences (Figure 2D).

**Figure 3.**
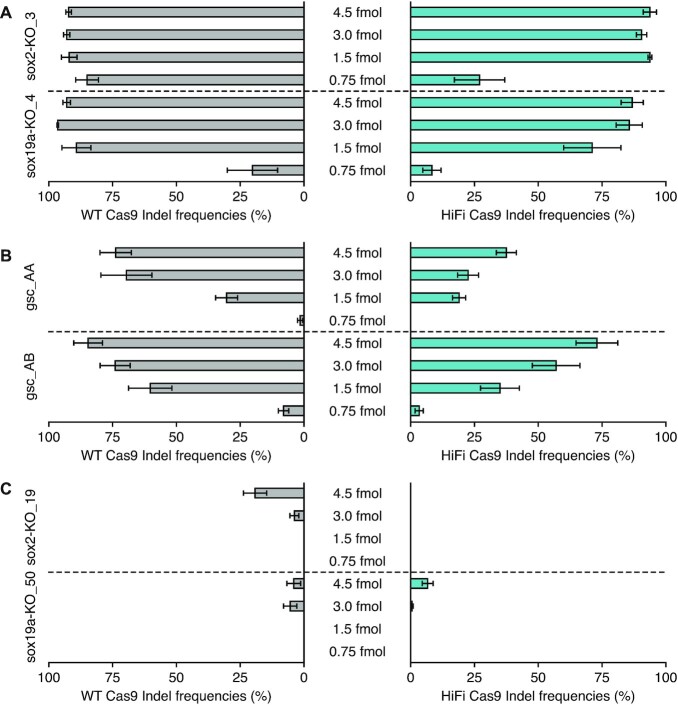
Dose-dependent cleavage efficiencies of selected crRNAs from low, medium, and high activity groups. Two crRNAs each from high (**A**), medium (**B**) and low (**C**) activity groups were injected as RNP at four different amounts of 0.75, 1.5, 3 and 4.5 fmol. Indel frequencies (in %) assessed with ICE for RNP complexes assembled with WT Cas9 and HiFi Cas9 are plotted on the left and right sides, respectively. Means of replicates are shown with standard error bars. The experimental dataset is shown in Supplementary Table S5.

We used sub-optimal conditions (a 1.5-fmol RNP injection) in the above experiments. It is an interesting question how decreased or increased amounts of RNP affect resulting indel frequencies. For this, we selected two crRNAs each from low, medium, and high activity groups and injected them as RNP at four different amounts of 0.75, 1.5, 3 and 4.5 fmol (Figure 3). crRNAs in the medium activity group exhibited varied indel frequencies depending on RNP amounts, whereas indel frequencies increased only slightly for the low activity group crRNAs, even at 4.5 fmol. Indel frequencies of sox2-KO_3 complexed with WT Cas9 reached 85%, even at 0.75 fmol, indicating its extremely high activity. These results together suggest that our strategy of using sub-optimal conditions successfully distinguished crRNA activities from low to high and that a more precise characterization might be achieved with the use of varying amounts of RNP.

### Assessment of cleavage efficiency of crRNAs with an *in vitro* cleavage assay

**Figure 4.**
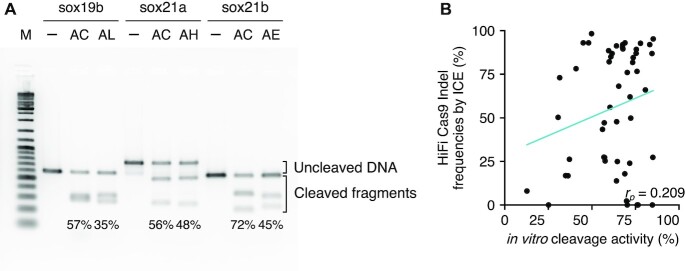
Assessment of cleavage efficiency of crRNA in an *in vitro* cleavage assay. (**A**) Representative image of agarose gel electrophoresis for the *in vitro* cleavage assay using HiFi Cas9. Uncleaved substrate fragments and cleaved fragments are indicated by brackets. Cleavage efficiencies for 51 crRNAs were calculated by quantifying band intensities of uncleaved fragments, as summarized in Supplementary Table S6. -, no RNP control; AC, AL, etc., suffixes of the respective crRNA names. (**B**) There was only weak correlation between cleavage efficiencies obtained with the *in vitro* cleavage assay and the ICE indel frequencies obtained by injection of HiFi Cas9 RNP into embryos. A scatter plot with a Pearson correlation coefficient (*r*_p_) is shown.

Potential advantages of the CRISPR-Cas9 RNP system may include applicability of an *in vitro* cleavage assay to assess cleavage efficiency before performing cell or embryo-based experiments. Thus, we also assessed cleavage efficiencies of 51 crRNAs in the form of HiFi Cas9 RNP complexes with the *in vitro* cleavage assay using purified PCR fragments as substrates under reaction conditions recommended by the supplier of CRISPR-Cas9 reagents (IDT). After incubation with CRISPR-Cas9 RNP, PCR fragments were separated on agarose gels and band intensities of uncleaved fragments were measured to calculate cleavage efficiencies (Figure 4A, Supplementary Table S6). To our surprise, cleavage efficiencies obtained using the *in vitro* cleavage reaction were only weakly correlated with those using the embryo assay (Pearson correlation coefficient, *r*_p_ = 0.209) (Figure 4B), although some reports have suggested that the *in vitro* cleavage assay is useful (51,52). These results suggest that at least under these conditions, the *in vitro* assay may have only limited utility for pre-screening of crRNAs for *in vivo* use.

### Identification of sequence features that determine crRNA efficiency

**Figure 5.**
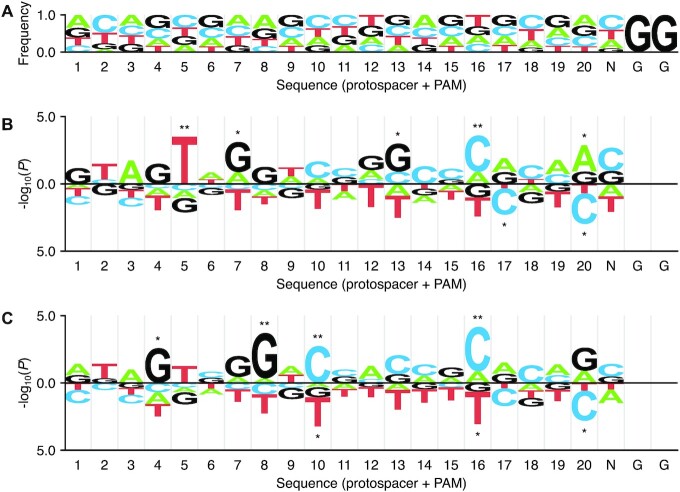
Position-specific mononucleotide features determined with kpLogo. (**A**) Nucleotide frequency at each position of input sequences. Position 1 is assigned to the 5′-terminal nucleotide of the 20-nt protospacer target sequence (positions 1–20) and positions 21–23 correspond to PAM. (**B**) A kpLogo plot of mononucleotide features obtained for WT Cas9. Favored and disfavored nucleotides are shown in the upper and lower sides, respectively, with the height scaled to its *P*-value (–log_10_ transformed) derived from Student's t tests of whether crRNAs with a particular nucleotide at a specific position were more or less efficient than other crRNAs. (C) A kpLogo plot of mononucleotide features obtained for HiFi Cas9. Significant nucleotides after Bonferroni correction done at each position are marked in (B) and (C) (**P < 0.01/4 = 0.0025; *P < 0.05/4 = 0.0125).

Several sgRNA-based studies have identified nucleotide features of the protospacer sequence that can affect CRISPR-Cas9 performance (12,14–26,46). To extract such sequence features for dgRNA-based CRISPR-Cas9 RNP, ICE indel frequency scores for 51 crRNAs were analyzed using the probability-based logo tool called kpLogo (k-mer probability logo) (46), which was used to identify gRNA sequence features in the original report. In the kpLogo program, protospacer and PAM sequences weighted by ICE scores were statistically evaluated, and then logo plots for position-specific mono- to tetranucleotide preference (1- to 4-letter k-mer motifs) were generated. Here, we focused on mononucleotide (*k* = 1) motifs due to the limited number of tested crRNAs. In these plots, at each position, mononucleotides were separately plotted vertically with the height scaled to the statistical significance [−log_10_(*P*-value)]. Despite the smaller sample size compared to previous studies, we obtained distinctive mononucleotide features for WT and HiFi Cas9 that were similar between WT and HiFi Cas9 (Figure 5). In this analysis, WT Cas9 exhibited clear positive and negative preferences for several positions: top five such features were 5-T(favored), which denotes that thymine was favored at position 5, 16-C(favored), 20-C(disfavored)/20-A(favored), 7-G(favored), 13-G(favored). HiFi Cas9 exhibited similar, but slightly distinct position-specific preference. The top five of these features were 16-C(favored)/16-T(disfavored), 8-G(favored), 10-C(favored)/10-T(disfavored), 4-G(favored), 20-C(disfavored)/20-G(favored), some of which were shared with WT Cas9 [e.g. 16-C(favored), 20-C(disfavored)]. Among these, 5-T, 8-G, 10-C and 13-G features appeared to affect cleavage efficiency to different degrees between WT and HiFi Cas9.

Interestingly, these position-specific mononucleotide features for WT Cas9 exhibited similarities to and differences from those obtained in previous sgRNA-based studies, particularly at the PAM-proximal and PAM-distal positions, respectively. Consistent with our observation, 20-G/A(favored) and 20-C(disfavored) have been repeatedly reported in previous sgRNA-based studies (12,14,16–18,22,24,32,46). 16-C(favored), which was identified for both WT and HiFi Cas9, has also been reported, albeit in a limited number of prior studies (14,22,46). In contrast, 4-G(favored), 5-T(favored), 7-G(favored) and 8-G(favored) were not evident in previous sgRNA-based studies, in which mononucleotide features in the PAM-distal region varied widely among them. One previous study reported that positions 1–14 are generally dominated by guanine in active sgRNAs in zebrafish (17), but our results indicate that the guanine preference appears to be highly position-dependent [e.g. 4-G(favored), 7-G(favored)]. Thymine is reportedly undesirable, particularly at the four nucleotide positions adjacent to PAM, since multiple uracils in sgRNA may cause low sgRNA expression driven by the U6 promoter (15,18,38). However, we still observed a bias against thymine throughout the protospacer, particularly at middle positions, suggesting that this thymine aversion may be intrinsic to CRISPR-Cas9.

Some previous studies have shown that GC content of the protospacer sequence affects cleavage efficiencies of sgRNAs (15,24,32,33), but others have reported conflicting results (23,53). In our data, the overall GC content of positions1-20 (35% - 80%) did not affect cleavage efficiencies of crRNAs (Supplementary Figure S1). When we examined the effects of sense and antisense strands targeted by crRNAs on cleavage efficiency, there was no statistically significant difference between them (Student's *P* = 0.261 for WT, *P* = 0.306 for HiFi) (Supplementary Figure S2), which is consistent with previous reports (14,53). Taken together, these results suggest that position-specific sequence features may be a primary determinant of crRNA activity.

### Experimental validation of sequence features associated with crRNA efficiency

**Figure 6.**
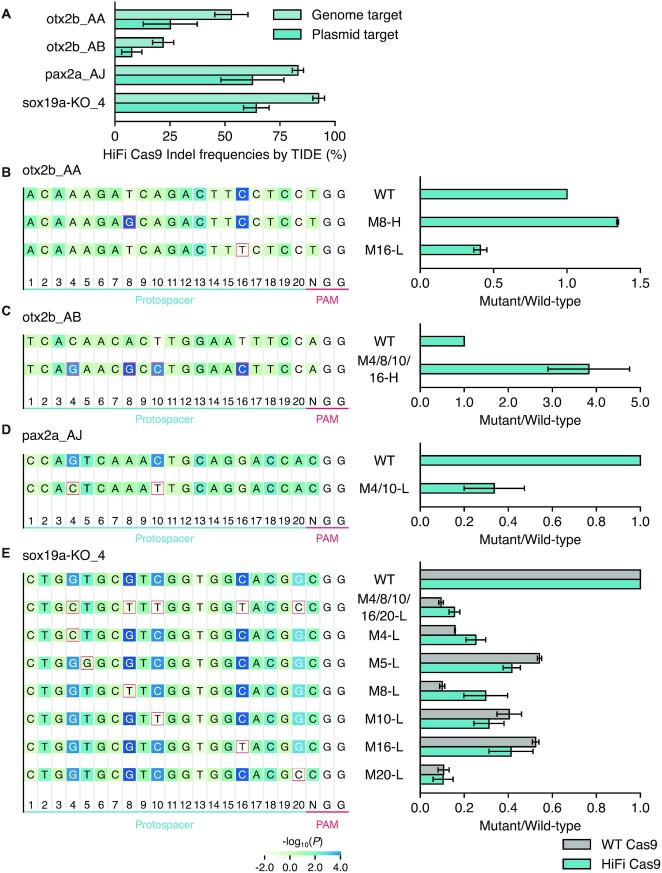
Experimental validation of mononucleotide features associated with crRNA efficiency. (**A**) Establishment of the *in vivo* plasmid assay. Plasmid substrates containing target sequences for otx2b_AA/AB, pax2a_AJ and sox19a-KO_4 crRNAs were first injected into 1-cell stage zebrafish embryos and corresponding HiFi Cas9 RNP complexes were subsequently injected to ensure *in vivo* cleavage reaction. Plasmid and genomic DNAs were extracted from isolated nuclei of injected embryos at 24 hpf for the TIDE analysis. Plasmid substrates were cleaved in a similar manner to genome targets, albeit less efficiently. Means of two injection experiments are shown with standard errors. (**B**−**D**) *In vivo* plasmid assays for mutant crRNAs of the otx2b_AA (B), otx2b_AB (C), and pax2a_AJ (D) using HiFi Cas9. In target sequences on the plasmid, nucleotides with high *P*-values were mutated into nucleotides with the opposite effect, as indicated by red squares. H and L denote mutations with disfavored to favored nucleotides and vice versa, respectively. Means of two injection experiments are shown as values relative to wild-type crRNA (Mutant/Wild-type), which were obtained by dividing indel frequencies of mutant plasmid targets induced by mutant crRNAs by those for corresponding wild-type crRNAs. Standard errors are also shown. (**E**) *In vivo* plasmid assays for mutant crRNAs of sox19a-KO_4 using WT Cas9 and HiFi Cas9. Means of two (WT Cas9) or three (HiFi Cas9) injection experiments are shown as values relative to WT crRNA with standard errors.

Although previous studies have identified position-specific sequence features of sgRNAs, as in the case of our study on crRNAs, their impact on cleavage efficiency has not been experimentally validated through mutagenesis, while maintaining a perfect match between crRNAs and targets. To evaluate effects of a specific nucleotide at a particular position, we mutated one or more nucleotides in the crRNA sequences from favored to disfavored nucleotides, or vice versa, based on our analysis, and examined their effects on cleavage efficiency in embryos. To this end, we developed a plasmid-based *in vivo* cleavage assay (called an *in vivo* plasmid assay hereafter) to measure cleavage efficiency of a mutant crRNA against the target sequence with the same mutation. We first cloned the target sequences of the otx2b_AA/AB, pax2a_AJ and sox19a-KO_4 crRNAs from the zebrafish genome as substrates for the *in vivo* plasmid assay and tested whether the plasmid substrates were cleaved similarly to the genomic targets. The plasmid substrate was first injected into 1-cell stage zebrafish embryos and the corresponding RNP complex was subsequently injected to ensure *in vivo* cleavage. Plasmid and genomic DNAs were extracted from isolated nuclei of injected embryos at 24 hpf. Interestingly, we were unable to detect enough cleavage on the plasmid targets without isolating nuclei by sucrose cushion centrifugation, which effectively removed plasmids in the cytoplasm. Even under these conditions, a double amount of CRISPR-Cas9 RNP was required to achieve plasmid target cleavage comparable to genome targets, albeit less efficiently. When we examined cleavage efficiencies of the four crRNAs under this condition, there was a good correlation between plasmid targets and genome targets (Figure 6A). Thus, this method was employed to evaluate cleavage efficiency of mutant crRNAs.

Here, we focused more on HiFi Cas9 mononucleotide features due to its importance for precise genome editing. We first used otx2b_AB, otx2b_AA and pax2a_AJ to represent crRNAs with low, moderate, and high cleavage efficiency, respectively, and examined the importance of four critical nucleotides, 4-G(favored), 8-G(favored), 10-C(favored), and 16-C(favored) on HiFi Cas9 activity by replacing these specific nucleotides with favored or disfavored nucleotides in the crRNA and corresponding target sequences (Figure 6B–D). When we compared relative cleavage efficiencies between wild-type crRNAs and mutant crRNAs, these mutations increased or decreased cleavage efficiencies as expected. Even the single-nucleotide mutations in otx2b_AA crRNA, 8-T(disfavored)→G(favored) and 16-C(favored)→T(disfavored), resulted in significant changes of cleavage efficiency (Figure 6B). Consistently, two nucleotide mutations in pax2a_AJ crRNA to disfavored nucleotides and the four nucleotide mutations at the four critical positions of otx2b_AB to favored nucleotides dramatically changed cleavage efficiency (Figure 6C and D). Then, we used sox19a-KO_4 crRNA as a highly active crRNA that has favored nucleotides at 5-T(favored) and 20-G(favored), in addition to the four tested positions, in order to examine the relative contribution of each mononucleotide feature to overall crRNA cleavage efficiency when complexed with both WT and HiFi Cas9. All single nucleotide mutations at any of positions 4, 5, 8, 10, 16 or 20 resulted in decreased cleavage activity to different degrees, indicating that these position-specific mononucleotide features all contributed to the full cleavage activity of sox19a-KO_4 crRNA (Figure 6E). Interestingly, among these mutations, the 20-G(favored)→C(disfavored) mutation resulted in the greatest reduction of cleavage activity. More interestingly, the mutation of PAM-distal 4-G(favored) to C(disfavored) had a higher impact on the cleavage efficiency of sox19a-KO_4 than PAM-proximal 16-C, suggesting that PAM-distal sequences are also important for high cleavage activity of CRISPR-Cas9, at least in the dgRNA/RNP system, although the PAM-proximal sequences (known as seed sequences) have been considered more important for sgRNA performance, particularly for target specificity. Taken together, this mutational analysis confirmed the importance of position-specific mononucleotide features identified through our crRNA evaluation and revealed their relative impact on cleavage efficiency.

### Predictive power of current CRISPR-Cas9 gRNA design tools for crRNAs

**Figure 7.**
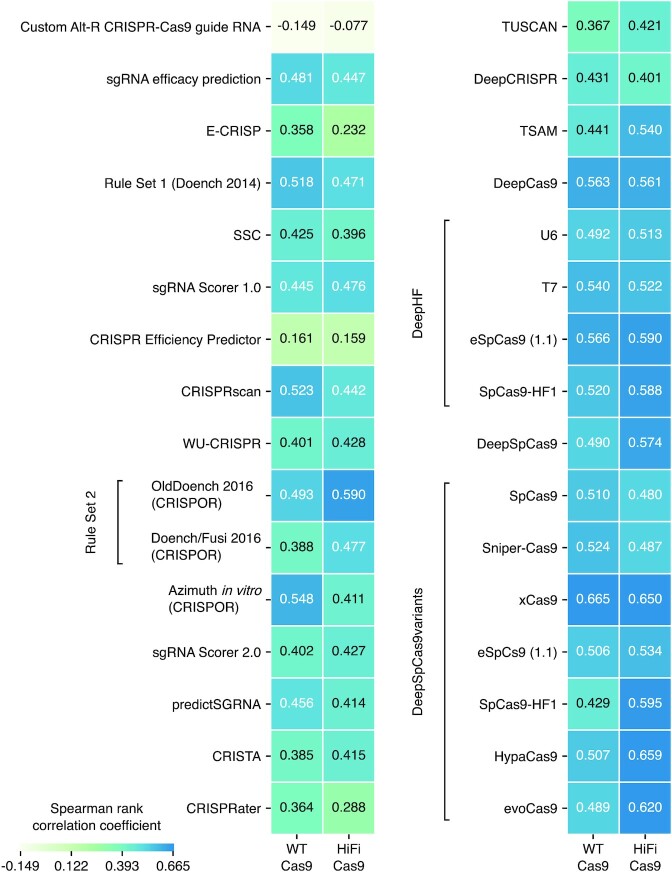
Predictive power of current CRISPR-Cas9 gRNA design tools for dgRNA/Cas9 RNPs. The heat map shows Spearman rank correlation coefficients (*r*_s_) between prediction scores of major gRNA design tools for 51 crRNAs tested in this study (Supplementary Tables S3 and S7) and ICE indel frequency values for WT (left) and HiFi (right) Cas9 (Supplementary Table S4). Corresponding scatter plots are shown in Supplementary Figure 3. For DeepSpCas9variants, tRNA-N20 sgRNA values were used.

Several gRNA prediction tools have been developed to pre-screen gRNA sequences *in silico* before *in vivo* use (12,14–23,26). As summarized in Supplementary Table S3, these are based on data from different experimental systems and prediction algorithms, but they are all based on experimental data using sgRNAs, except for the IDT design tool. Using the major gRNA design tools, we obtained prediction scores for 51 crRNAs tested in this study (Supplementary Table S7) and compared them with the ICE indel frequency scores of WT and HiFi Cas9 RNPs (scatter plots are shown in Supplementary Figure S3). When Spearman rank correlation coefficients between actual and predicted values were calculated, these gRNA design tools predicted crRNA activity with different degrees of precision (Figure 7). ICE values unexpectedly exhibited little correlation with on-target scores derived from the IDT design tool, although it was presumably developed with data from cultured cell-based experiments using dgRNA-based RNP. In contrast, traditional tools, including Rule Set 1 (Doench) and CRISPRscan, and deep-learning-based tools, including DeepCas9, DeepHF, and DeepSpCas9variants, predicted cleavage efficiencies of crRNAs with relatively high coefficients for both WT and HiFi Cas9 (Figure 7), suggesting that current sgRNA-based gRNA design tools are able to predict cleavage efficiency of crRNAs in dgRNA-based RNP to some extent. The relatively higher predictive power of CRISPRscan that has been developed based on the zebrafish dataset using *in vitro* transcribed sgRNA may be attributed to use of the same experimental animal model (17). DeepCas9, DeepHF, and DeepSpCas9variants used deep-learning-based computational models for prediction, suggesting an advantage of deep learning-based algorithms. However, DeepSpCas9variants for xCas9, not for WT Cas9, outputted the most predictive values for our WT Cas9 data, suggesting that the gRNA datasets themselves may affect the predictive power more than the modelling approach.

### Prediction of gRNA efficiency by position-specific mononucleotide feature-based scoring

Since we successfully identified position-specific mononucleotide features linked to probability values, we examined whether these could be used to predict efficiency of gRNAs as suggested by the kpLogo manual (46). A similar strategy was also reported in a previous *Drosophila* cell-based study with some success (23). In calculating probability value-based scores (we call them CRISPR-kp scores), the respective [−log_10_(*P*-value)] values were summed, such that negative values were given for disfavored features. When the calculation was applied to the original 51 crRNAs, scores ranged from −15.1 to + 19.9 and from −14.3 to + 21.1 for WT and HiFi Cas9, respectively (Supplementary Table S8).

**Figure 8.**
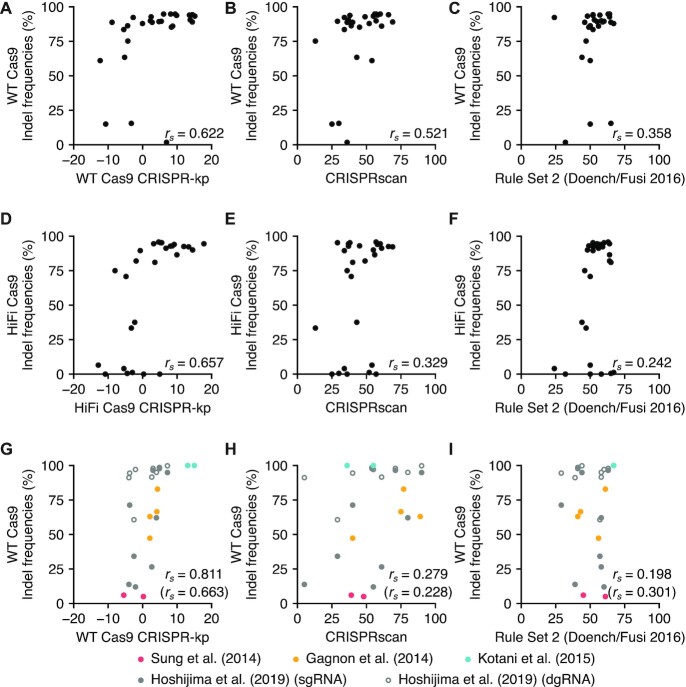
Prediction of gRNA efficiency by CRISPR-kp, position-specific mononucleotide feature-based scoring. (**A**−**F**) Performance of CRISPR-kp on independent crRNA efficiency data in comparison with CRISPRscan and Rule Set 2. CRISPR-kp scores were obtained by summing the respective mononucleotide feature [−log_10_(*P*-value)] values, in which negative values were given for disfavored mononucleotide features (Supplementary Table S8). Cleavage efficiencies of the second set of 27 crRNAs were determined using the dgRNA RNP complex assembled with WT or HiFi Cas9 with ICE (Supplementary Figure S4). Indel frequencies (in %) obtained with WT Cas9 (A−C) and HiFi Cas9 (D−F) were compared to scores obtained with CRISPR-kp (A,D), CRISPRscan (B,E) and Rule Set 2 (Doench/Fusi 2016) (C, F) by scatter plots. (**G**−**I**) Performance of CRISPR-kp on published data in comparison with CRISPRscan and Rule Set 2. Reported gRNA efficiencies assessed with RNP injections into zebrafish embryos (9,24,54,55) are compared with their CRISPR-kp (G), CRISPRscan (H), and Rule Set 2 (Doench/Fusi 2016) (I) scores. gRNA sequences and their CRISPR-kp scores are shown in Supplementary Table S8. Spearman correlation coefficients (*r*_s_) are indicated in each panel. In panels G-I, Spearman correlation coefficients for data including Hoshijima's dgRNAs are shown in parentheses.

To examine whether CRISPR-kp scores correlate with activity of other crRNAs, we designed a second set of 27 crRNAs, which were selected to have varied CRISPR-kp scores (Supplementary Table S8). These crRNAs were assessed for their cleavage efficiencies using both WT and HiFi Cas9 (Supplementary Figure S4 and Supplementary Table S9). We observed high correlation between CRISPR-kp scores and indel frequencies, particularly for HiFi Cas9 (Figure 8A and D). Spearman rank correlation coefficients of CRISPR-kp scores were higher than those of CRISPRscan, which was developed for zebrafish sgRNAs (17) and Rule set 2 (Doench/Fusi 2016 score), which has been most used for prediction of U6-based sgRNAs (19) (Figure 8B, C, E and F).

To further validate CRISPR-kp, we next examined whether CRISPR-kp scores correlated with gRNA efficiencies reported in previous zebrafish studies performed under conditions similar to ours, in which CRISPR-Cas9 RNP was injected into zebrafish embryos. For this, we generated scores for sgRNAs used in the four publications (9,24,54,55) (Supplementary Table S8). Again, CRISPR-kp scores of the combined dataset correlated well with reported indel frequencies (Figure 8G). Although Hoshijima *et al.* (9) tested both sgRNAs and dgRNAs, correlation with the dataset including Hoshijima's sgRNAs was better than that for dgRNAs, probably because the use of larger amounts of CRISPR-Cas9 RNP resulted in very high activity of almost all dgRNAs, whereas activity of sgRNAs was decreased to comparable levels due to supernumerary G at the 5′-end of *in vitro* transcribed sgRNAs. These correlations were greater than those with CRISPRscan scores and the Rule set 2 (Doench/Fusi 2016) scores (Figure 8H and I). In contrast, indel frequencies derived from lipofection delivery of CRISPR-Cas9 RNP to cultured cells (39) exhibited little correlation with CRISPR-kp scores (Supplementary Figure S5), suggesting that the RNP delivery method may critically affect gRNA efficiency. Taken together, these results suggest that probability value-based CRISPR-kp scores may be useful for gRNA design, particularly when sgRNA/crRNAs are to be used as a component of CRISPR-Cas9 RNP and delivered by microinjection.

### Distinct profiles of dgRNA efficiency between injection of pre-assembled RNP and a dgRNA/Cas9 mRNA mixture

**Figure 9.**
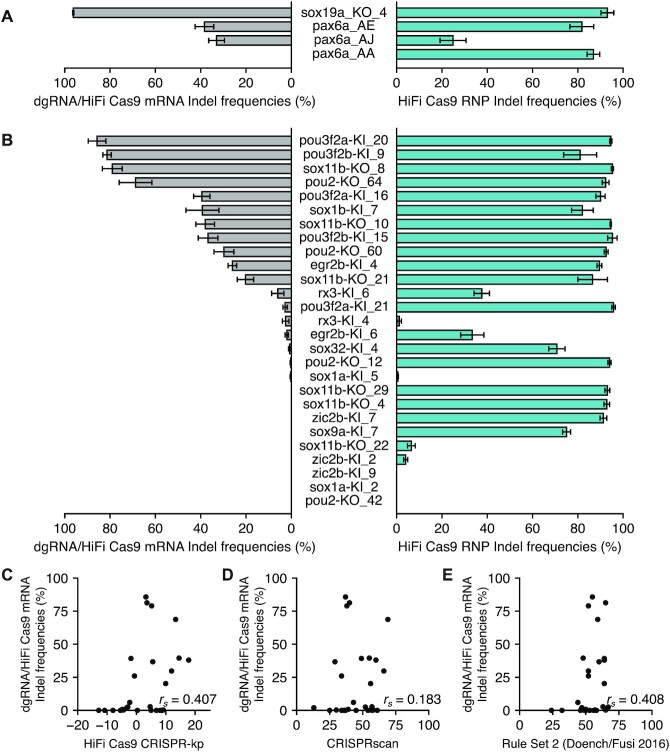
Comparison of dgRNA efficiency between injection of pre-assembled RNP and a dgRNA/Cas9 mRNA mixture. (**A**) Selected crRNAs from the first set of 51 crRNAs were tested for their activity through injection of 3 fmol dgRNA with 200 pg HiFi Cas9 mRNA. Indel frequencies (in %) assessed with ICE were compared to those obtained with HiFi Cas9 RNP, using bar graphs. Means of replicates are shown with standard error bars. The experimental dataset for dgRNA/Cas9 mRNA mixture is shown in Supplementary Table S9. HiFi Cas9 RNP data were adopted from Figure 2. (**B**) A second set of 27 crRNAs were tested for their activity through injection of 3 fmol dgRNA with 200 pg HiFi Cas9 mRNA. Indel frequencies (in %) assessed with ICE were compared to those obtained with HiFi Cas9 RNP using bar graphs. Means of replicates are shown with standard error bars. The experimental dataset for dgRNA/Cas9 mRNA mixture is shown in Supplementary Table S9. HiFi Cas9 RNP data were adopted from Supplementary Figure S4. (C−E) Performance of CRISPR-kp on efficiency of 27 crRNAs in dgRNA/HiFi Cas9 mRNA format in comparison with CRISPRscan and Rule Set 2 scores. Indel frequencies (in %) obtained with dgRNA/HiFi Cas9 mRNA were compared to scores obtained with CRISPR-kp (**C**), CRISPRscan (**D**) and Rule Set 2 (Doench/Fusi 2016) (**E**) using scatter plots. Spearman correlation coefficients (*r*_s_) are indicated in each panel.

Efficiency of gRNAs is reportedly affected by their RNA stability and interaction with Cas9, which relates to RNP complex formation efficiency, when they are delivered with Cas9 mRNA (15,17,24). A recent study (56) indicates that complete RNP complex formation can be achieved when dgRNA and Cas9 protein are mixed in a 1:1 ratio *in vitro*, as done in our study, whereas efficiency of *in vivo* RNP complex formation is unpredictable when Cas9 mRNA is used. Based on preliminary experiments, we found that for some crRNAs, injection of 3 fmol dgRNA with 200 pg HiFi Cas9 mRNA resulted in indel frequencies comparable to those by 1.5-fmol RNP, but other crRNAs exhibited lower activity (Figure 9A), suggesting that dgRNA efficiency may not be the same between injection of pre-assembled RNP and dgRNA/Cas9 mRNA mixture, as reported in a previous study (24). To further clarify this point, we examined cleavage efficiency of a second set of 27 crRNAs using HiFi Cas9 mRNA. Consistently, the majority of crRNAs exhibited lower cleavage efficiencies, when compared to those by RNP (Figure 9B). Several crRNAs with high activity in RNP format exhibited drastically decreased or no cleavage activity in dgRNA/Cas9 mRNA format, which results in moderate correlation between RNP and mRNA indel frequencies (Pearson correlation coefficient, *r*_p_ = 0.505). Accordingly, correlation with CRISPR-kp scores was also decreased (Spearman correlation coefficient, *r*_s_ = 0.407) (Figure 9C), similar to that with Rule set 2 scores (Figure 9E). Taken together, these results suggest that when CRISPR-Cas9 components are delivered as RNAs and assembled into RNP *in vivo*, gRNA efficiency could be strongly influenced by sequence features that govern RNA stability and RNP complex formation efficiency.

## DISCUSSION

In this study, we identified sequence features of crRNA that critically affect activity of dgRNA-based CRISPR-Cas9 in the form of pre-assembled RNP complexes for WT Cas9, as well as HiFi Cas9. To the best of our knowledge, this is the first systematic attempt to identify such sequence features for dgRNA-based CRISPR-Cas9 RNP. Successful identification of these features may be attributable to advantages of our approach. First, the use of *in vitro* assembled RNP complexes eliminated several problems associated with sgRNA expression systems. *In vivo* expression of sgRNAs from the U6 promoter could be variable, because Pol III may cause premature termination when sgRNAs contain U-rich sequences (15,38). Moreover, G enrichment and A depletion in sgRNA sequences increase their stability and interaction with Cas9 could affect RNP complex formation, which in turn affects their efficiency (15,17). This point is further supported by the fact that striking differences of indel frequencies were observed between injection of dgRNA/Cas9 RNP and dgRNA along with Cas9 mRNA (Figure 9). Furthermore, use of RNA polymerase for sgRNA expression regardless of *in vivo* or *in vitro* expression often requires mismatched or supernumerary G at the 5′-end of sgRNAs (5,6,9,24). These ambiguous factors may hamper accurate evaluation of sequence features that affect sgRNA cleavage efficiency. Second, the delivery method of RNP complexes, that is, microinjection into zebrafish embryos, allowed us to precisely control the amount of CRISPR-Cas9, which is important to measure gRNA efficiency in the optimal range, as shown by our dose-response experiment (Figure 3). Third, the use of zebrafish embryos could reduce the effect of chromatin state on CRISPR-Cas9 activity that is observed for differentiated cultured cells (34,37). Prior to maternal to zygotic transition (MZT), the genome is undergoing continuous and rapid replication and DNA synthesis, which could increase the chance of CRISPR-Cas9-mediated cleavage. Moreover, zebrafish embryos lack apparent heterochromatin, characterized by histone H3 lysine 9 trimethylation (H3K9me3) and condensed chromatin ultrastructure (57), suggesting that the effects of chromatin states may be minimal in pre-MZT embryos. Consistently, cleavage activities of the crRNAs targeting the same gene exhibited considerable variation, suggesting that crRNA sequences themselves have the greatest impact on their efficiency. More importantly, we found that our CRISPR-kp scores derived from probability values linked to mononucleotide features correlated well with indel frequencies reported in zebrafish experiments using CRISPR-Cas9 RNP, as well as our cleavage efficiency data of the independent crRNA set (Figure 8). These together suggest that gRNA efficiency is primarily determined by its sequence when pre-assembled with Cas9 protein. On the other hand, a drawback of our strategy is scale limitation. Synthetic crRNAs are more costly than *in vitro* transcribed gRNAs and injection into zebrafish embryos is time consuming, which makes high throughput difficult.

Position-specific nucleotide features for crRNAs exhibited more similarities than differences between WT and HiFi Cas9 (Figure 5). This is consistent with previous findings that HiFi Cas9 exhibits on-target activity comparable to WT Cas9, albeit slightly less efficiently, while increasing cleavage specificity, regardless of delivery methods (RNP complexes with dgRNA or *in vivo* expression with sgRNA) (39,45). This characteristic is unique to HiFi Cas9 among engineered high-fidelity Cas9 variants, since other variants such as SpCas9-HF1 and eSpCas9 (1.1) have reduced on-target activity when delivered as RNP complexes (39), suggesting an advantage of HiFi Cas9 for high-specificity genome editing in zebrafish, where the pre-assembled RNP is easily delivered by microinjection. However, there were a few exceptional crRNAs that exhibited high cleavage efficiency with WT Cas9, but were inactive with HiFi Cas9 (e.g. sox1a-KI_2, rx3-KI_4 and zic2b-KI_2) (Supplementary Figure S4), suggesting that testing WT Cas9 would be beneficial when a particular crRNA fails to induce indels with HiFi Cas9.

Position-specific nucleotide features for crRNA in the form of pre-assembled RNP complexes exhibited some similarities to those determined with sgRNA systems using U6 or T7 promoter, particularly in the PAM-proximal region. Most previous studies identified 20-C(disfavored)/20-G(favored) with high significance (12,14,16–18,22,24,32,46), which was verified experimentally through the mutational analysis in this study. This preference is likely linked to the fact that a nucleotide at position 20 is important for Cas9-induced PAM-dependent DNA melting and RNA−DNA hybrid formation, which was revealed by the crystal structure analysis of the sgRNA-Cas9 complex (58). The preference for 16-C(favored) has also been identified in several reports (14,22,46) and its importance was again experimentally verified in this study. In contrast, clear differences were observed at PAM-distal positions. Particularly, the preference for 4G and 5T has not been reported in sgRNA-based studies, suggesting that these features may be specific to the condition in which a perfectly matched 20-nucleotide sequence is used for gRNA and/or the use of pre-assembled RNP. Furthermore, our mutational analysis revealed that 4-G(favored) and 5-T(favored) had a similar or even higher impact on cleavage activity than those in the PAM-proximal region. For other positions that exhibited lower statistical significance, a more systematic mutational analysis might reveal their contribution to overall crRNA efficiency.

When we compared the predictive power of current gRNA design tools using our on-target cleavage efficiency data, considerable variation was observed among them. A previous study suggested that the on-target prediction model strongly depends on the sgRNA expression systems used in the experiments in which the original dataset was created, in which either the U6 promoter for *in vivo* transcription or the T7 promoter for *in vitro* transcription was employed (20). Consistent with this, our data suggest that gRNA stability and structure and the CRISPR-Cas9 delivery system may affect evaluation of gRNA efficiency because we observed striking differences of indel frequencies between injection of dgRNA/Cas9 RNP and co-injection of dgRNA with Cas9 mRNA (Figure 9). In fact, our probability value-based CRISPR-kp scores exhibited clear correlations with indel frequencies of the independent set of 27 crRNAs as well as indel frequencies that were reported in zebrafish injection experiments using CRISPR-Cas9 RNP (Figure 8). In contrast, these measured and reported indel frequencies exhibited lower correlations with scores derived from CRISPRscan based on data using injection of T7-derived sgRNA/Cas9 mRNA and Rule set 2 (Doench/Fusi 2016) based on data using U6-promoter sgRNA/PolII-promoter Cas9. On the other hand, the correlation decreased when CRISPR-Cas9 RNP was delivered into cultured cells by lipofection, suggesting that the RNP delivery system may also critically affect gRNA efficiency. This may explain why the IDT design tool that was likely developed using lipofection data yielded scores divergent from all other design tools (Figure 7). Therefore, the applicability of a particular gRNA design tool is influenced by the experimental conditions used for gRNA efficiency evaluation and CRISPR-kp scores may best fit with the CRISPR-Cas9 RNP injection system. CRISPR-kp scores of a crRNA of interest for WT and HiFi Cas9 can be obtained using the Excel spreadsheets (Supplementary Table S8) by entering its protospacer plus PAM sequence. As expected by design, positive scores suggest high probability of successful cleavage.

One of the interesting findings of this study is that *in vitro* cleavage efficiencies did not correlate well with *in vivo* activity (Figure 4). Similarly, CRISPR-Cas9 mismatch tolerance reportedly shows distinct patterns between *in vitro* and *S. cerevisiae* (59). There are many variables that could affect *in vitro* CRISPR-Cas9 activity, which include buffer composition, incubation temperature, and substrate complexity (e.g. genomic DNA versus PCR amplicons) (60). On the other hand, *in vivo* mutagenesis rates would not faithfully reflect *in vitro* CRISPR-Cas9 activity, since repair of Cas9-induced breaks tends to be error prone, but error rates depend on the locus (61). Therefore, further studies are required to clarify the causes of these discrepancies.

In conclusion, we determined the mononucleotide sequence features of crRNA that critically affect activity of dgRNA-based CRISPR-Cas9 in the form of pre-assembled RNP complexes for HiFi Cas9, as well as WT Cas9. As discussed above, these features may be largely free from effects of gRNA stability and gRNA loading efficiency into Cas9. These features exhibited similarities to those determined with sgRNA systems to some extent, particularly in the PAM-proximal region, but not in the PAM-distal region, suggesting that these PAM-distal features may be specific to perfectly matched gRNAs and/or the use of pre-assembled RNP. Based on these sequence features, we developed a probability value-based CRISPR-kp score, which will be beneficial for crRNA design in future genome editing studies, particularly those employing injection of CRISPR-Cas9 RNP.

## DATA AVAILABILITY

Probability value-based CRISPR-kp scores for crRNAs of interest can be obtained using the Excel spreadsheets (Supplementary Table S8) by replacing protospacer plus PAM sequences of the crRNAs.

## Supplementary Material

gkac100_Supplemental_Files
