# Supplementary material for: Key sequence features of CRISPR RNA for dual-guide CRISPR-Cas9 ribonucleoprotein complexes assembled with wild-type or HiFi Cas9
Source: Nucleic Acids Res. 2022 Feb 15;50(5):2854–71. doi: 10.1093/nar/gkac100 (PMC8934663; doi:10.1093/nar/gkac100)
Supplement: gkac100_Supplemental_Files [file gkac100_supplemental_files.zip › Okada_Supplementary-Figures.pdf]

Supplementary Material

Key sequence features of CRISPR RNA for dual-guide CRISPR-Cas9 ribonucleoprotein complexes assembled with wild-type or HiFi Cas9

Keita Okada[1], Kanae Aoki[1], Teruyuki Tabei[1], Kota Sugio[1], Katsunori Imai[1], Yuki Bonkohara[1], and Yusuke Kamachi[1,*]

[1] School of Environmental Science and Engineering, Kochi University of Technology, Kami, Kochi 782-8502, Japan

* To whom correspondence should be addressed. Tel: 81-887-57-2510; Fax: 81-887-57-2520; Email: kamachi.yusuke@kochi-tech.ac.jp

Supplementary Figure S1. Correlation between GC content of crRNAs and indel frequencies.

Supplementary Figure S2. Correlation between strands targeted by crRNAs and indel frequencies.

Supplementary Figure S3. Correlation between gRNA design tool scores and indel frequencies.

Supplementary Figure S4. Cleavage efficiencies of the second set of 27 crRNAs in the dgRNA RNP complex assembled with WT or HiFi Cas9.

Supplementary Figure S5. Performance of CRISPR-kp on dgRNAs used in RNP lipofection.

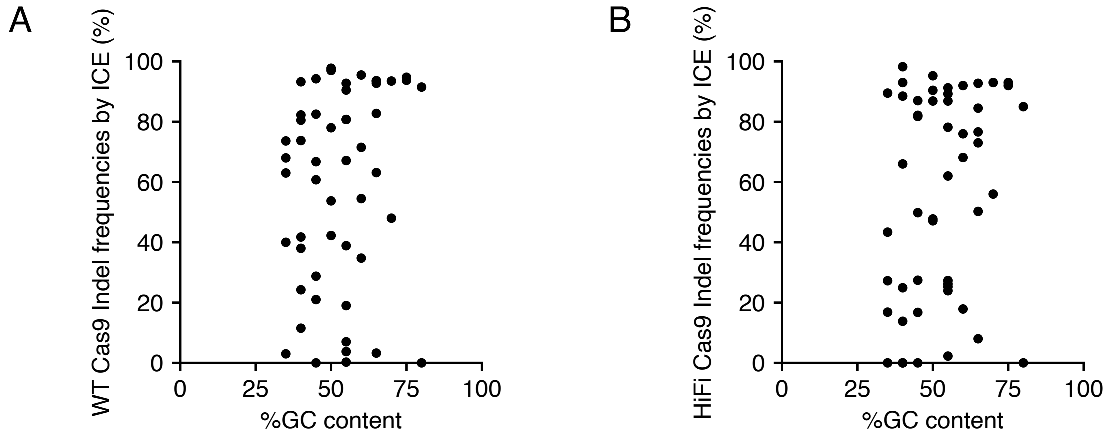

Supplementary Figure S1. Correlation between GC content of crRNAs and indel frequencies. Comparison of indel frequencies (in %) generated with WT Cas9 RNP (A) and HiFi Cas9 RNP (B) with GC content of the protospacer sequences (in %).

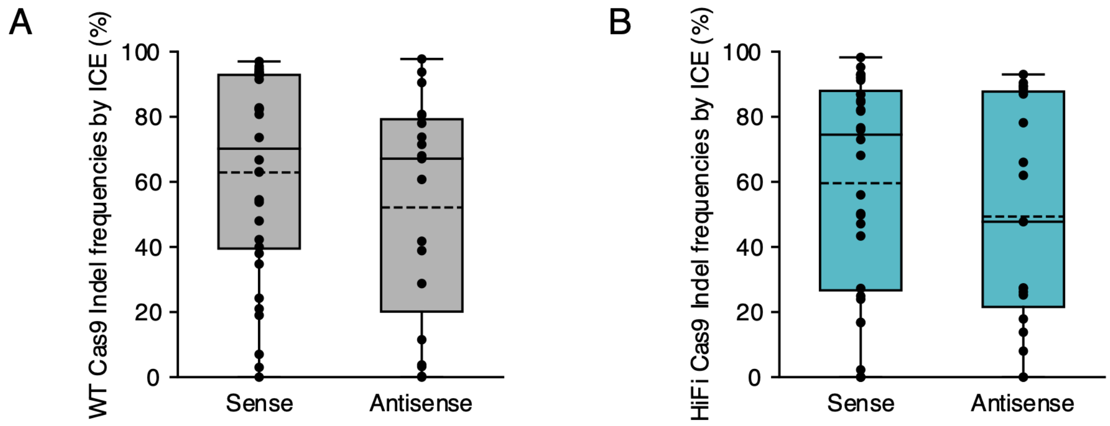

Supplementary Figure S2. Correlation between strands targeted by crRNAs and indel frequencies. The target strand of crRNAs corresponds to the strand that is bound by the crRNAs. Comparison of indel frequencies (in %) generated by WT Cas9 RNP (A) and HiFi Cas9 RNP (B) with the strand targeted by crRNAs. Box-and-whisker plots show ranges and 25th percentile/median/75th percentile. Means are indicated by dotted lines.

A

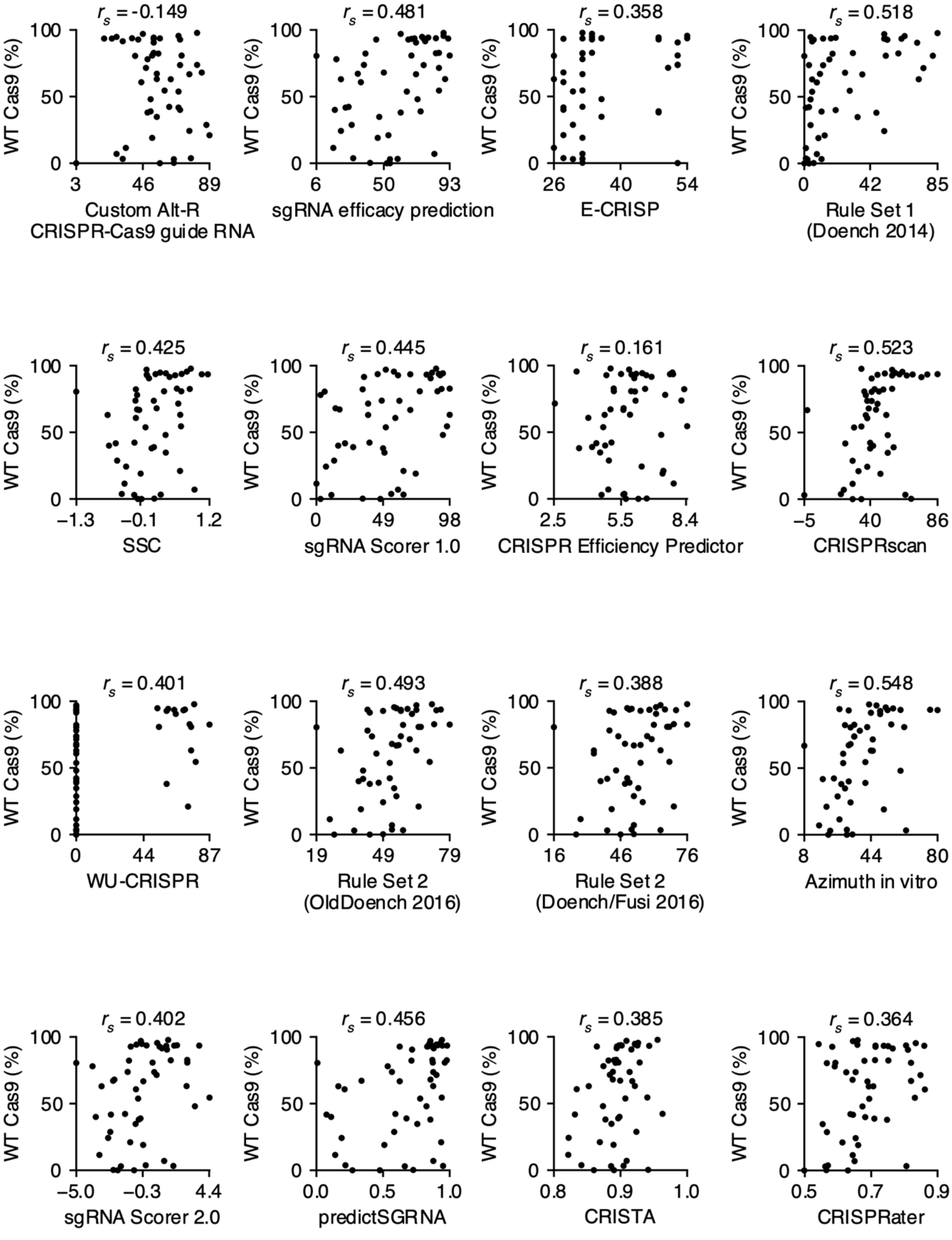
r_s = -0.149
r_s = 0.481
r_s = 0.358
r_s = 0.518
WT Cas9 (%)
Custom Alt-R CRISPR-Cas9 guide RNA
sgRNA efficacy prediction
E-CRISP
Rule Set 1 (Doench 2014)
r_s = 0.425
r_s = 0.445
r_s = 0.161
r_s = 0.523
SSC
sgRNA Scorer 1.0
CRISPR Efficiency Predictor
CRISPRscan
r_s = 0.401
r_s = 0.493
r_s = 0.388
r_s = 0.548
WU-CRISPR
Rule Set 2 (OldDoench 2016)
Rule Set 2 (Doench/Fusi 2016)
Azimuth in vitro
r_s = 0.402
r_s = 0.456
r_s = 0.385
r_s = 0.364
sgRNA Scorer 2.0
predictSGRNA
CRISTA
CRISPRater

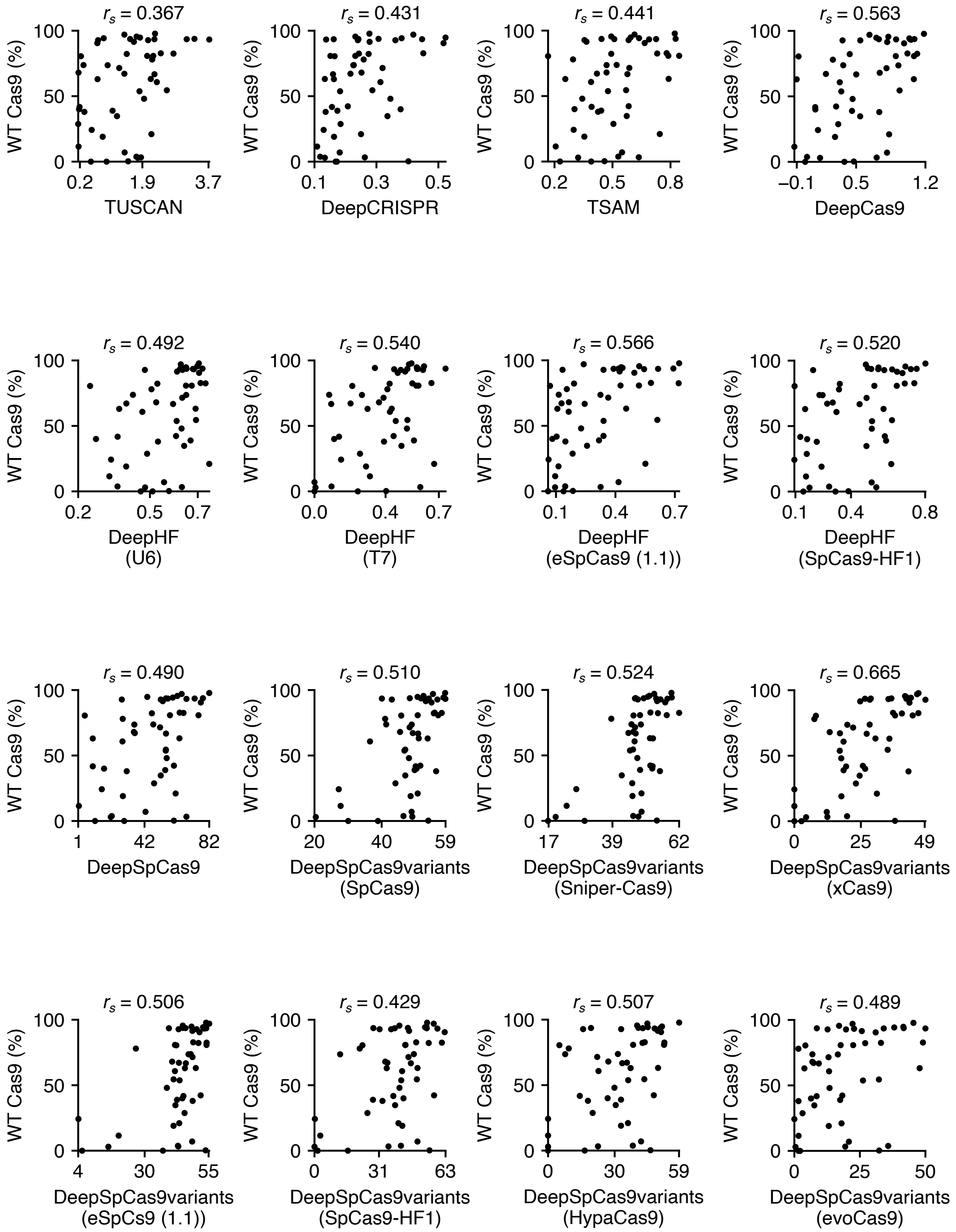

r_s = 0.367
WT Cas9 (%)
TUSCAN
r_s = 0.431
DeepCRISPR
r_s = 0.441
TSAM
r_s = 0.563
DeepCas9
r_s = 0.492
DeepHF (U6)
r_s = 0.540
DeepHF (T7)
r_s = 0.566
DeepHF (eSpCas9 (1.1))
r_s = 0.520
DeepHF (SpCas9-HF1)
r_s = 0.490
DeepSpCas9
r_s = 0.510
DeepSpCas9variants (SpCas9)
r_s = 0.524
DeepSpCas9variants (Sniper-Cas9)
r_s = 0.665
DeepSpCas9variants (xCas9)
r_s = 0.506
DeepSpCas9variants (eSpCs9 (1.1))
r_s = 0.429
DeepSpCas9variants (SpCas9-HF1)
r_s = 0.507
DeepSpCas9variants (HypaCas9)
r_s = 0.489
DeepSpCas9variants (evoCas9)

B

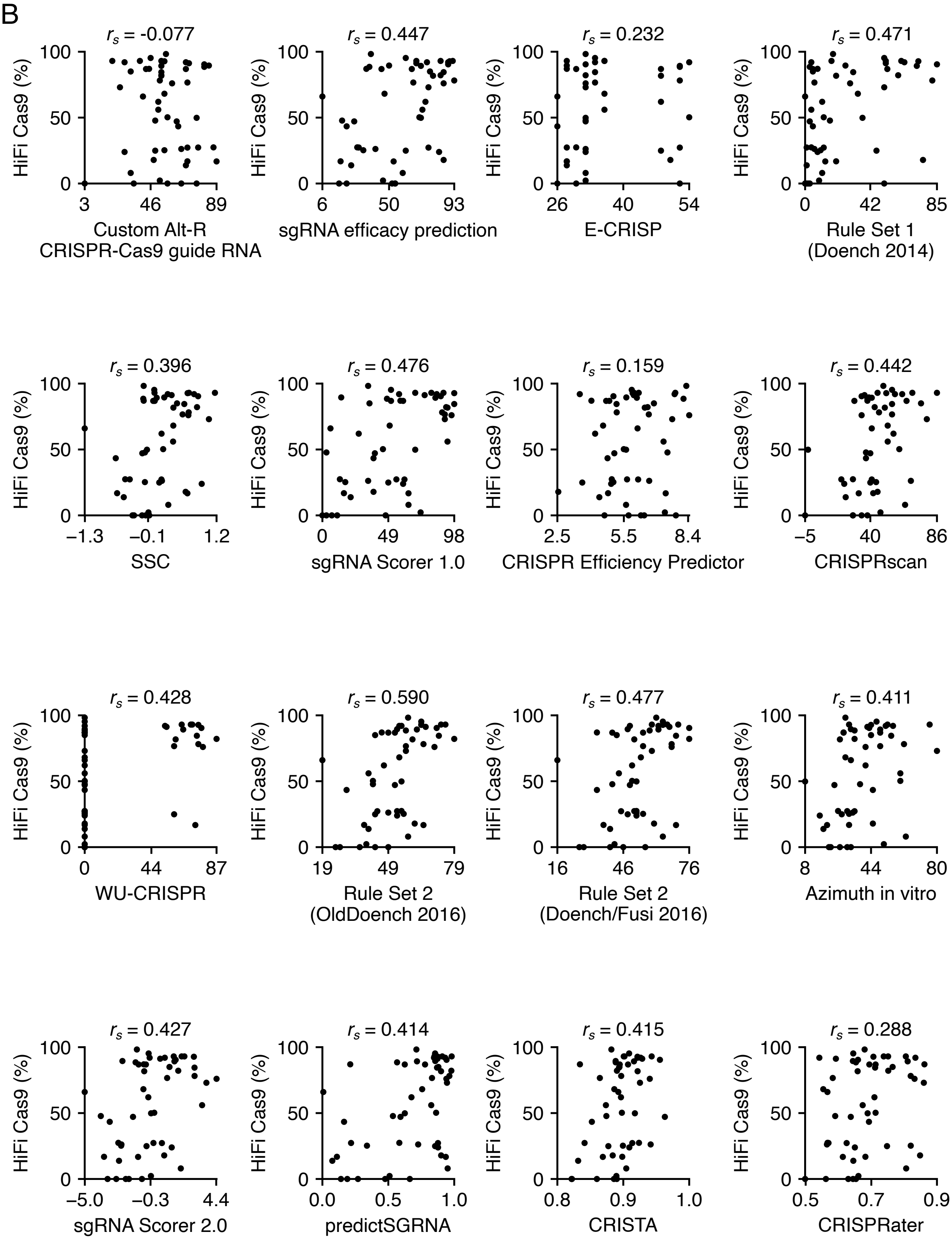

r_s = -0.077
HiFi Cas9 (%)
100
50
0
3
46
89
Custom Alt-R CRISPR-Cas9 guide RNA
r_s = 0.447
6
50
93
sgRNA efficacy prediction
r_s = 0.232
26
40
54
E-CRISP
r_s = 0.471
0
42
85
Rule Set 1 (Doench 2014)
r_s = 0.396
−1.3
−0.1
1.2
SSC
r_s = 0.476
0
49
98
sgRNA Scorer 1.0
r_s = 0.159
2.5
5.5
8.4
CRISPR Efficiency Predictor
r_s = 0.442
−5
40
86
CRISPRscan
r_s = 0.428
0
44
87
WU-CRISPR
r_s = 0.590
19
49
79
Rule Set 2 (OldDoench 2016)
r_s = 0.477
16
46
76
Rule Set 2 (Doench/Fusi 2016)
r_s = 0.411
8
44
80
Azimuth in vitro
r_s = 0.427
−5.0
−0.3
4.4
sgRNA Scorer 2.0
r_s = 0.414
0.0
0.5
1.0
predictSGRNA
r_s = 0.415
0.8
0.9
1.0
CRISTA
r_s = 0.288
0.5
0.7
0.9
CRISPRater

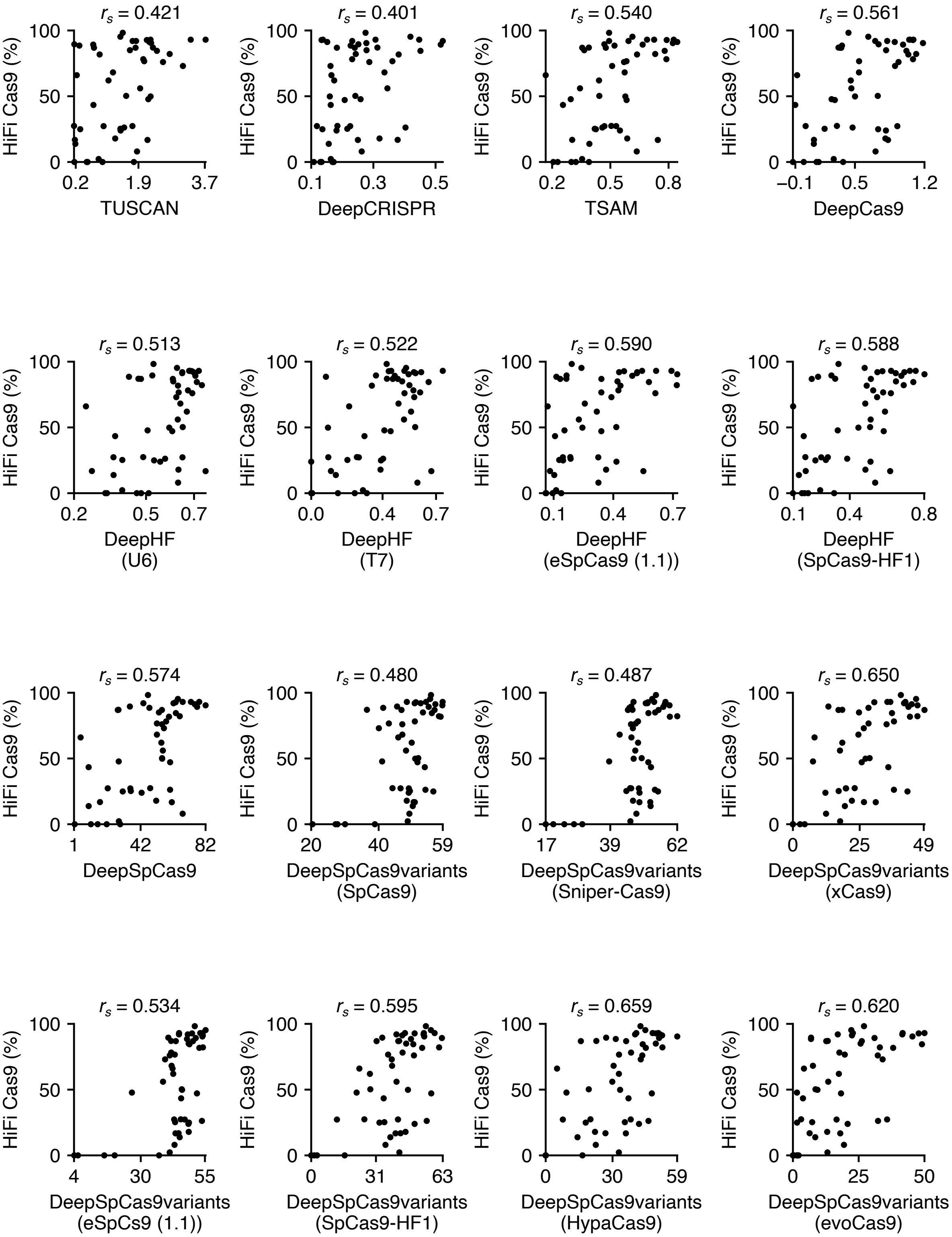

Supplementary Figure S3. Correlation between gRNA design tool scores and indel frequencies. Scatter plots between predicted efficiency scores and indel frequencies that were generated by WT Cas9 RNP (A) and HiFi Cas9 RNP (B) and determined with ICE are shown. Spearman rank correlation coefficients (r_s) are indicated in each panel.

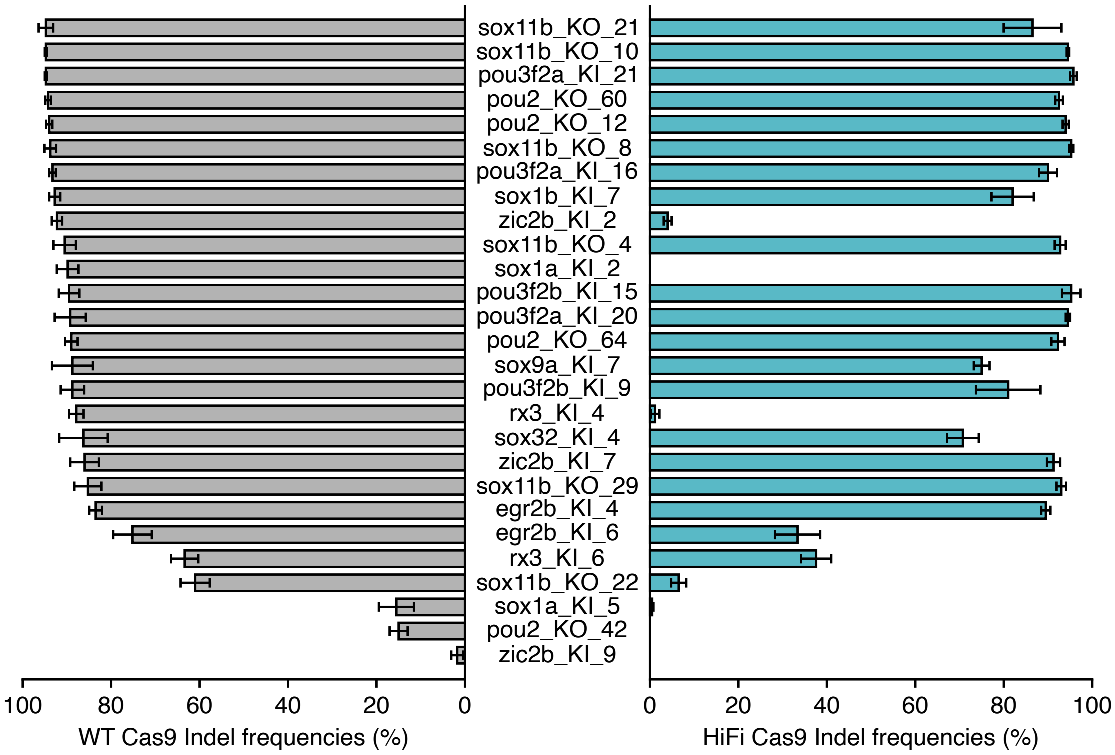

Supplementary Figure S4. Cleavage efficiencies of the second set of 27 crRNAs in the dgRNA RNP complex assembled with WT or HiFi Cas9. Indel frequencies (in %) assessed with ICE for the second set of 27 crRNAs in dgRNA RNP complexes assembled with WT Cas9 and HiFi Cas9 are plotted on the left and right sides, respectively. Means of replicates are shown with standard error bars. The experimental dataset is shown in Supplementary Table S9.

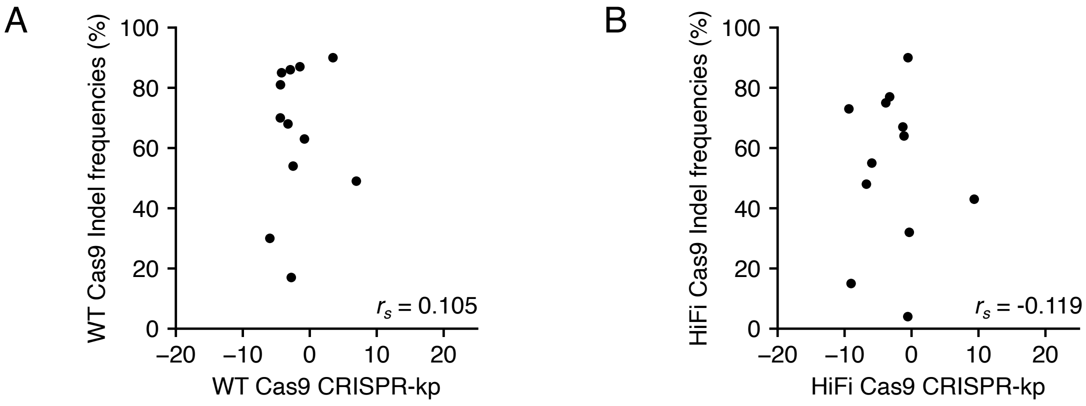

Supplementary Figure S5. Performance of CRISPR-kp on dgRNAs used in RNP lipofection. Reported dgRNA efficiencies assessed by RNP lipofection into cultured cells (39) are compared with CRISPR-kp scores. (A) WT Cas9. (B) HiFi Cas9. Spearman correlation coefficients (r_s) are indicated in each panel.
